# Bladder necrosis following uterine artery embolization for post‐abortion uterine arteriovenous malformation: A rare complication

**DOI:** 10.1002/ijgo.70589

**Published:** 2025-10-10

**Authors:** Mira Zlotnik, Marcos de Lorenzo Messina, Eduardo Zlotnik, José Maria Soares Júnior, Edmund Chada Baracat

**Affiliations:** ^1^ Department of Obstetrics and Gynecology, Faculdade de Medicina, Hospital das Clínicas Universidade de São Paulo (HCFMUSP) São Paulo São Paulo Brazil; ^2^ Hospital Israelita Albert Einstein São Paulo São Paulo Brazil

**Keywords:** bladder necrosis, manual vacuum aspiration, postabortion complication, uterine arteriovenous malformation, uterine artery embolization

Uterine arteriovenous malformations are uncommon but potentially serious causes of abnormal uterine bleeding, often acquired after procedures such as curettage, manual vacuum aspiration (MVA), or uterine surgery.^1^ Uterine artery embolization (UAE) is an effective, minimally invasive treatment, offering high success rates while preserving fertility.^2^ However, complications such as uterine necrosis have been reported, while bladder involvement remains extremely rare.^3^, ^4^

We report the case of a 35‐year‐old woman (G2P1A1) with reproductive desire who underwent MVA for abortion. She developed profuse vaginal bleeding and anemia. Two months after the MVA, she was diagnosed with a uterine arteriovenous malformation. Pelvic angiography confirmed uterine arteriovenous fistula, with nidus on the right (Figure 1a). Catheterization of the right uterine artery revealed a patent, tortuous vessel with parenchymal blush. Superselective embolization was performed using ethylene vinyl alcohol copolymer. Persistent perfusion via the right cervical artery required additional embolization using the same agent. The left uterine artery was similarly involved and was embolized using Histoacryl diluted in Lipiodol after superselective catheterization (Figure 1b).

**FIGURE 1:**
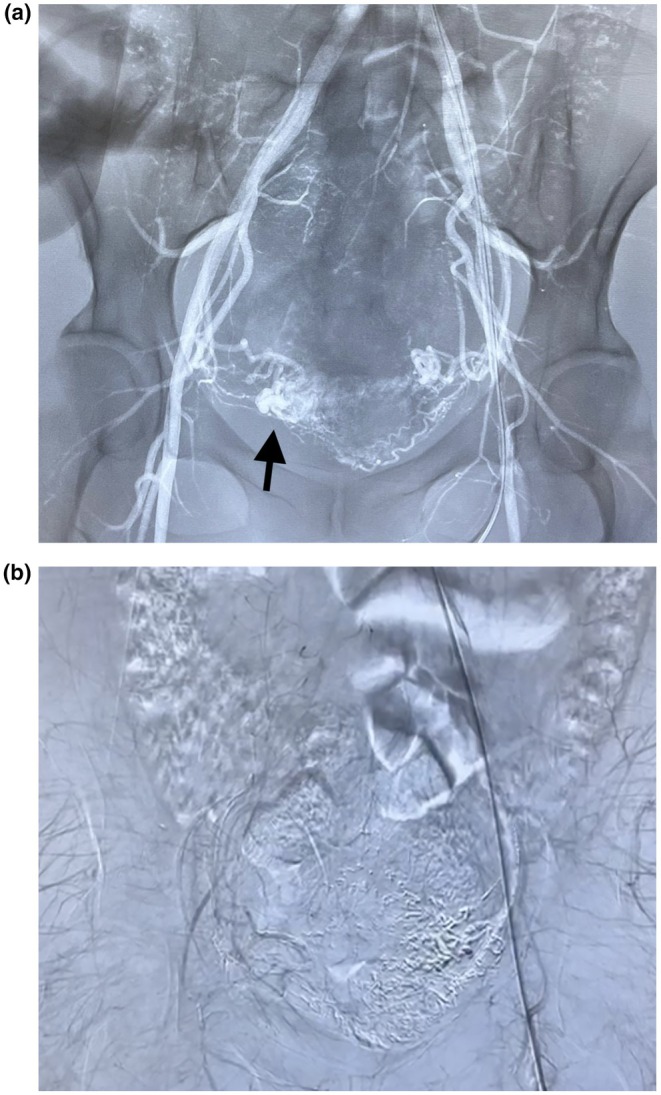
Digital subtraction angiography during uterine artery embolization. (a) Preembolization angiography demonstrating markedly tortuous uterine arteries supplying an arteriovenous fistula with early venous drainage and a dense nidus on the right side (arrow). (b) Postembolization control image demonstrating successful occlusion of the arteriovenous fistula following bilateral embolization.

One month after embolization she returned to the hospital with abdominal pain and hematuria. Magnetic resonance imaging raised the hypothesis of fistula as it revealed a 1.3‐cm defect in the posterior bladder wall communicating with the embolized uterine region. Cystoscopy revealed a well‐demarcated 4‐cm necrotic area with central ulceration, consistent with bladder wall necrosis (Figure 2). Instillation of methylene blue with a vaginal tampon was negative for vesicouterine fistula. It was managed conservatively with a Foley catheter for 3 weeks. Follow‐up cystoscopy showed healing mucosa. Hormonal blockade with a gonadotropin‐releasing hormone analog was initiated for persistent light bleeding, which was effective, and the patient remains in amenorrhea. Follow‐up magnetic resonance imaging demonstrated no abnormalities in the uterus or bladder.

**FIGURE 2:**
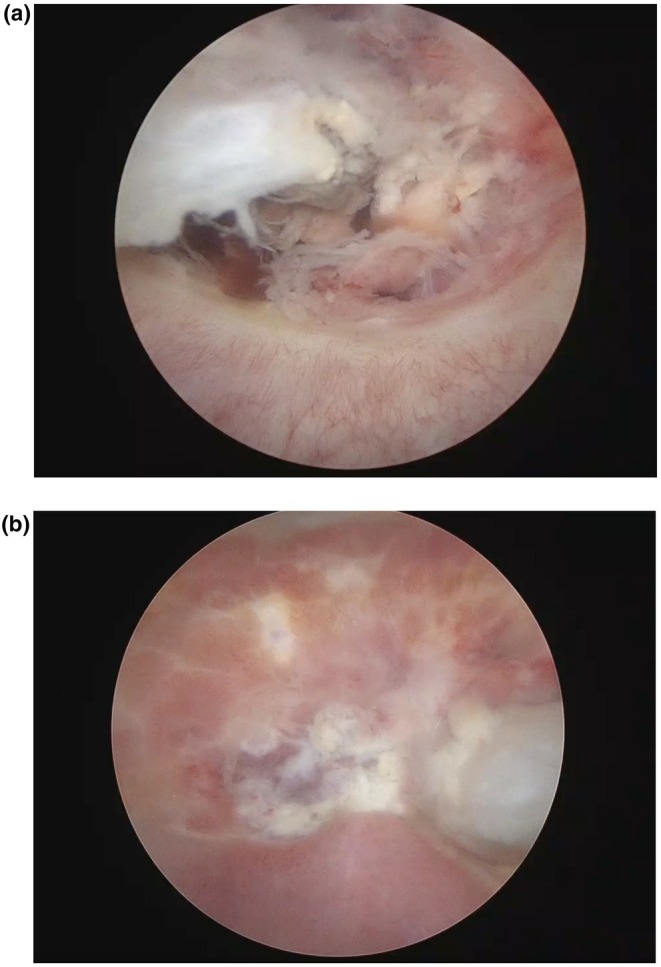
Cystoscopic views of the bladder wall during diagnostic evaluation 1 month after uterine artery embolization. (a) Bladder base with a large, pale, friable necrotic area (≈4 cm) with central mucosal ulceration. (b) Adjacent bladder wall with irregular necrotic mucosa and hyperemic changes, consistent with ischemic injury.

Bladder necrosis following UAE is exceptionally rare, especially without uterine involvement. Conservative management proved successful in this case, avoiding surgical reconstruction while preserving fertility potential. Early diagnosis is important for successful preservation of organs by adopting conservative management when the patient's hemodynamic status is stable.^3^, ^4^ This case emphasizes the importance of recognizing rare complications of UAE and demonstrates that conservative management can be successful. Written informed consent was obtained from the patient for publication of this case report in accordance with institutional ethics guidelines.

## AUTHOR CONTRIBUTIONS

Mira Zlotnik: Conceptualization; investigation; writing—original draft; writing—review and editing. Marcos de Lorenzo Messina and Eduardo Zlotnik: Investigation; data analysis; writing—review and editing. José Maria Soares Júnior and Edmund Chada Baracat: Supervision; writing—review and editing; final approval.

## FUNDING INFORMATION

None.

## CONFLICT OF INTEREST STATEMENT

The authors have no conflicts of interest.

## Data Availability

Data sharing is not applicable to this article as no new data were created or analyzed in this study.
